# Real-Time Culture-Independent Microbial Profiling Onboard the International Space Station Using Nanopore Sequencing

**DOI:** 10.3390/genes12010106

**Published:** 2021-01-16

**Authors:** Sarah Stahl-Rommel, Miten Jain, Hang N. Nguyen, Richard R. Arnold, Serena M. Aunon-Chancellor, Gretta Marie Sharp, Christian L. Castro, Kristen K. John, Sissel Juul, Daniel J. Turner, David Stoddart, Benedict Paten, Mark Akeson, Aaron S. Burton, Sarah L. Castro-Wallace

**Affiliations:** 1 JES Tech, Houston, TX 77058, USA; sarah.e.stahl@nasa.gov (S.S.-R.); hang.n.nguyen@nasa.gov (H.N.N.); christian.l.castro@nasa.gov (C.L.C.); 2 UCSC Genomics Institute, University of California, Santa Cruz, CA 95064, USA; miten@soe.ucsc.edu (M.J.); benedict@soe.ucsc.edu (B.P.); makeson@soe.ucsc.edu (M.A.); 3 Astronaut Office, NASA Johnson Space Center, Houston, TX 77058, USA; richard.r.arnold@nasa.gov (R.R.A.); serena.m.aunon@nasa.gov (S.M.A.-C.); 4 KBR, Houston, TX 77058, USA; gretta.m.sharp@nasa.gov; 5 Project Management and Systems Engineering Division, NASA Johnson Space Center, Houston, TX 77058, USA; kristen.k.john@nasa.gov; 6 Oxford Nanopore Technologies, New York, NY 10013, USA; Sissel.Juul@nanoporetech.com; 7 Oxford Nanopore Technologies, Oxford Science Park, Oxford OX4 4DQ, UK; daniel.turner@nanoporetech.com (D.J.T.); david.stoddart@nanoporetech.com (D.S.); 8 Astromaterials Research and Exploration Science Division, NASA Johnson Space Center, Houston, TX 77058, USA; aaron.burton@nasa.gov; 9 Biomedical Research and Environmental Sciences Division, NASA Johnson Space Center, Houston, TX 77058, USA

**Keywords:** nanopore sequencing, in-situ analysis, field-deployable methods, bacterial identification, spaceflight

## Abstract

For the past two decades, microbial monitoring of the International Space Station (ISS) has relied on culture-dependent methods that require return to Earth for analysis. This has a number of limitations, with the most significant being bias towards the detection of culturable organisms and the inherent delay between sample collection and ground-based analysis. In recent years, portable and easy-to-use molecular-based tools, such as Oxford Nanopore Technologies’ MinION™ sequencer and miniPCR bio’s miniPCR™ thermal cycler, have been validated onboard the ISS. Here, we report on the development, validation, and implementation of a swab-to-sequencer method that provides a culture-independent solution to real-time microbial profiling onboard the ISS. Method development focused on analysis of swabs collected in a low-biomass environment with limited facility resources and stringent controls on allowed processes and reagents. ISS-optimized procedures included enzymatic DNA extraction from a swab tip, bead-based purifications, altered buffers, and the use of miniPCR and the MinION. Validation was conducted through extensive ground-based assessments comparing current standard culture-dependent and newly developed culture-independent methods. Similar microbial distributions were observed between the two methods; however, as expected, the culture-independent data revealed microbial profiles with greater diversity. Protocol optimization and verification was established during NASA Extreme Environment Mission Operations (NEEMO) analog missions 21 and 22, respectively. Unique microbial profiles obtained from analog testing validated the swab-to-sequencer method in an extreme environment. Finally, four independent swab-to-sequencer experiments were conducted onboard the ISS by two crewmembers. Microorganisms identified from ISS swabs were consistent with historical culture-based data, and primarily consisted of commonly observed human-associated microbes. This simplified method has been streamlined for high ease-of-use for a non-trained crew to complete in an extreme environment, thereby enabling environmental and human health diagnostics in real-time as future missions take us beyond low-Earth orbit.

## 1. Introduction

Microbial monitoring of crewed spacecraft has been an important aspect of infectious disease mitigation since early crewed missions [1,2,3,4,5]. Many engineering controls are implemented specifically to reduce the microbial burden in the habitable volume of space vehicles. For example, on the International Space Station (ISS), all surface materials must be cleanable to a given level of colony forming units (CFU), high-efficiency particulate air (HEPA) filtration is required for the air system, and the Water Processor Assembly (WPA) has mechanisms to reduce microbial contamination, including catalytic oxidation, filtration, and the addition of a biocide [1,2]. Despite these preventative measures, microorganisms cannot, and should not, be completely eliminated from the spaceflight environment. To assess the efficiency of these engineering controls and to monitor the levels of opportunistic pathogens in the environment, crewmembers sample the ISS air, water, and surfaces on a quarterly basis [1,6]. NASA’s current microbial monitoring requirements for crewed spacecraft habitability involve both quantification and identification of cultured bacteria and fungi. With quantitative information, NASA microbiologists can determine if the areas sampled meet the microbial acceptability limits, and these assessments can be completed in-flight in consultation with ground support [1,7]. While this quantitative information is useful, arguably more important in assessing microbial risk to the crew is the identification of the organisms present. Currently, the identity of the cultured microbes remains unknown until the samples are returned to the ground for analysis. The average time from sample collection by the crew onboard the ISS to when the microbes are returned to the laboratory on the ground and identified can range from weeks to months. While remediation of areas exceeding the CFU limits can occur within roughly a week of sample collection, remediation due to the presence of organisms that could present a risk to the vehicle or crew takes substantially longer.

A significant step towards rapid, in situ identification was achieved in August of 2017 when, for the first time, microbes were collected, cultured, and identified onboard the ISS [8]. Building on previous demonstrations of the molecular capabilities of miniPCR (miniPCR bio^TM^, Cambridge, MA, USA) and the MinION (Oxford Nanopore Technologies (ONT), Oxford, UK) [8,9,10], astronaut Peggy Whitson performed in-flight sample preparation and 16S amplicon sequencing, enabling the identification of *Staphylococcus hominis* and *Staphylococcus capitis* from Tryptic Soy Agar (TSA) slides collected as part of the nominal quarterly monitoring [8]. While this changed the paradigm of spaceflight microbial monitoring, many hurdles still exist around culture-dependent processes that must be cleared prior to exploration-class missions. Inherent issues in the spaceflight environment with culture dependent methods include the time needed for cultivation, the potential to expose the crew to high levels of pathogens, and the exclusion of important but non-culturable organisms. With human exploration taking aim at a return to the Moon and a venture to Mars, there is a critical need for rapid, safe, and thorough microbial monitoring capabilities.

With culture-independent molecular assays increasing our understanding of microbiomes on Earth and in space [11,12,13,14] and with the proven spaceflight capabilities of miniPCR and the MinION [8,9,10], a spaceflight-compatible swab-to-sequencer method was designed and subjected to ground-based laboratory and field testing before being transitioned for evaluation onboard the ISS. Ground-based testing was conducted specifically with regard to the NASA Microbiology Laboratory’s standard operating procedures (SOPs) for microbial identification. Each ground location was sampled with two swabs, with one being processed using the current culture and Sanger sequencing methods and the other subjected to the swab-to-sequencer nanopore-based method. Among the goals were to determine if the data from the swab-to-sequencer were sufficient for NASAs microbial risk assessment and if any important culturable isolates were lacking from the nanopore method. Analog field-testing during NASA’s Extreme Environment Mission Operations (NEEMO) 21st and 22nd missions provided method validation for use in extreme environments and improvements in streamlining procedures to enable any crew to perform successfully. Onboard the ISS, four independent swab-to-sequencer experiments were conducted by two different crewmembers. The astronauts collected surface swabs, extracted the DNA, performed bead-based purifications, 16S amplification, library preparation, and nanopore sequencing all onboard the ISS in support of the Biomolecule Extraction and Sequencing Technology (BEST) payload. This simplified sequencing method provides a means of culture-independent microbial monitoring onboard the ISS, which is a critical advancement for monitoring crew health in real-time; the results of these laboratory, NEEMO, and ISS-based analyses are reported here.

## 2. Materials and Methods 

### 2.1. Culture-Independent, Swab-to-Sequencer Method

**Figure 1:**
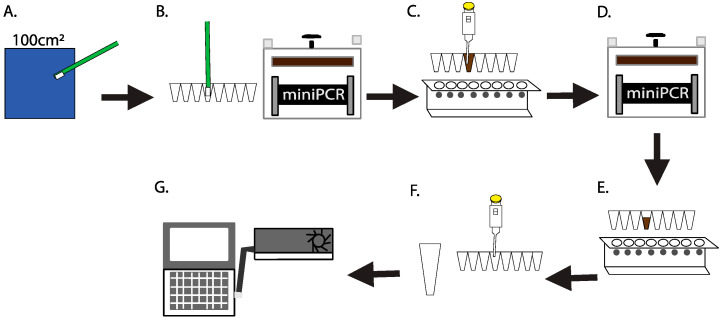
Workflow of the in-situ culture-independent, swab-to-sequencer process for extreme environment microbial profiling. (**A**) Swabbing of a 100 cm^2^ area. (**B**) Insertion of the swab head directly into lysis buffer in a PCR tube and placement into miniPCR for DNA extraction. (**C**) Bead-based DNA purification (**D**) DNA amplification within miniPCR. (**E**) Bead-based PCR amplicon purification. (**F**) Addition of sequencing control DNA and ONT-based rapid adaptor library preparation. (**G**) Flow cell loading and MinION sequencing.

Sterile packaged swabs (Texwipe, Kernersville, NC, USA; Idaho Technology, Inc. Salt Lake City, UT, USA), along with a 1.5-mL tube of sterile nuclease-free molecular grade water (Corning, Tewksbury, MA, USA) and a PCR tube strip containing 200 µL of QuickExtract™ DNA Extraction Solution (Lucigen Cooperation, Middleton, WI, USA) in each tube were taken to the sampling location. A swab was then aseptically removed from the packaging, wetted with sterile molecular grade water, and a 100 cm^2^ area was swabbed for 60 s using a rolling and rotating motion (Figure 1A). To collect the negative control, a swab was aseptically wetted with sterile water, but not touched to any surface. The swab tip was then immediately broken off into the DNA extraction solution and placed into the miniPCR thermal cycler (miniPCR bio, Cambridge, MA, USA) for DNA extraction at 65 °C for 15 min and 98 °C for 2 min (Figure 1B). Extracted DNA was purified using a 1X Agencourt AMPure XP bead (Beckman Coulter Genomics, Brea, CA, USA) cleanup. Washing buffer was modified to use 5% polyethylene glycol containing 1.25 M sodium chloride in place of ethanol. The DNA was eluted in 25 µL of sterile nuclease-free molecular grade water (Figure 1C). All laboratory ground studies were quantified using Qubit 1x dsDNA high sensitivity kit following the manufacturer’s protocol (Thermo Fisher Scientific, Waltham, MA, USA). A volume of 20 µL of purified DNA was added to PCR tubes containing ONT 16S barcoded primers (ONT modified 27F and 1492R, SQK-RAB204, ONT), LongAmp^®^ Taq 2X Master Mix (New England Biolabs (NEB), Ipswich, MA, USA) and sterile molecular grade water. The strip of tubes was placed into miniPCR and the reaction was carried out using the following conditions: initial denaturation at 95 °C for 180 s, 30 cycles of 95 °C for 20 s, 55 °C for 30 s and 65 °C for 120 s with a final extension at 65 °C for 300 s (Figure 1D).

The PCR product was purified using a 0.6X Agencourt AMPure XP bead cleanup with the modified washing buffer described above and eluted with 10 μL of 10 mM Tris-HCl pH 8.0 with 50 mM NaCl (Figure 1E). This bead clean-up ratio was recommended by Oxford Nanopore Technologies to remove primers, primer-dimers or spurious amplicons and select for the target amplicons (~1500 bp). Prior to sample loading, flow cell quality and total pore availability were determined following ONT procedures. The flow cell was washed through two additions of 200 μL flush buffer and flush tether mixture (ONT) through the sample loading port. As a positive control for the library preparation and sequencing, 5 ng of a ~720 bp *Apis mellifera* amplicon compatible with the ONT sequencing adaptor was added to the 16S amplicons (ONT). The combined amplicons were added to 1 μL of Rapid Adaptor (ONT) in 4 μL of additional Rapid Adaptor storage buffer (ONT) and incubated for 5 min at ambient temperature (Figure 1F). Following incubation, the library was added to Sequencing Buffer (ONT) and 150 μL was injected into the sample port of the flow cell. Sequencing was initiated on the MinKNOW software and was performed for up to 48 h (Figure 1G).

### 2.2. Comparison of Current Culture-Dependent to Swab-to-Sequencer, Culture-Independent Methods

Two swabs held in tandem were used to collect a surface sample following the same swabbing process as described above from each sample site at the NASA Johnson Space Center’s (JSC) Human Health and Performance Laboratory (Houston, TX, USA) (Supplementary Table S1). A total of 60 swabs were used to collect samples from 23 locations (46 total swabs) with the remaining 7 sets of swabs (14 total) serving as negative controls. After sample collection, one swab was directly inserted into the PCR tubes containing DNA Extraction Solution using the method described above. The second swab was placed into 3 mL of Tryptic Soy Broth (TSB, Hardy Diagnostics, Santa Maria, CA, USA) for processing following the NASA JSC Microbiology Laboratory’s SOPs for bacterial identification. Following this SOP allows for assessment between our swab-to-sequencer method and the standard culture-based method that has been historically used for ISS samples. Briefly, the TSB containing the swab was vortexed for 1 min and 100 µL was plated on TSA and incubated at 35 °C for 48 h to allow for enumeration and isolation. All colonies were individually selected and streaked onto TSA and cultured for 24 h to obtain pure isolates. DNA was extracted from pure colonies using the PrepMan^TM^ Ultra kit per the manufacturer’s protocol (ThermoFisher Scientific, Waltham, MA, USA). The first 500 bp of the 16S rDNA gene was amplified and prepared for sequencing using the MicroSEQ 500 16S rDNA PCR containing primer 5F and 531R [15] and Sequencing Kits and sequenced using an ABI 3500 Genetic Analyzer (ThermoFisher Scientific). Analysis was completed using the MicroSEQ ID 3.0 software (ThermoFisher Scientific).

### 2.3. NASA Analog Operations

In June 2017, on the ocean floor within the Aquarius Reef Base, crewmembers supporting the 22nd NEEMO mission tested a preliminary version of the swab-to-sequencer protocol described above with minor modifications. During the mission, crewmembers remain isolated in and around the Aquarius habitat, located 5.6 km off the coast of Key Largo, FL at a depth of 19 m for 10 days [16]. Its small size (15 m × 4.6 m) and isolated extreme location provides an ideal ISS analog for training crew and testing new science procedures and hardware development [17]. All supplies necessary for testing were assembled into a portable kit and all reagents were packaged into one-time-use tubes and color-coded for ease-of-use by the crew. Supplies were delivered to the Aquarius habitat via the Research Vessel Sabina where the Aquarius Support Team loaded the supplies into sealed metal pots and swam them to Aquarius. Cold reagents were transported on ice and, once inside Aquarius, were placed in a Yeti Tundra cooler filled with five 1-quart ice blocks. The ice blocks were replaced daily to try to maintain cold conditions. Environmental conditions within Aquarius averaged 24 °C ambient temperature with 2.5 atm atmospheric pressure [16,17].

A list of swabbing locations was suggested by the science team, with actual sampling sites being determined by crew preference. Swabs were collected, and DNA was extracted and purified as described above. DNA was amplified and prepared for sequencing using the SQK-RAS201 16S Rapid Amplicon Sequencing Kit (ONT) following a modified manufacturer’s protocol. The conditions for PCR were set up as follows: initial denaturation at 95 °C for 300 s, 30 cycles of 94 °C for 10 s, 55 °C for 30 s and 65 °C for 75 s and a final extension at 65 °C for 300 s. PCR products were cleaned up and prepared as described above. Sequence ready DNA was added to 120 µl running buffer and 80 µl nuclease-free water and loaded into a primed FLO-MAP107 R7 flow cell. The NC_48Hr_Sequencing_Run_FLO-MIN106_SQK-RAS201_plus_Basecaller.py protocol was initiated in the MinKNOW software (Version 1.6.1.1) and sequencing runs proceeded for at least 11 h for all runs.

### 2.4. Spaceflight Hardware

The Biomolecule Extraction and Sequencing Technology (BEST) payload utilized a mini8 miniPCR thermal cycler (miniPCR bio), a MinION sequencer (ONT), and Eppendorf Research Plus pipettes (Eppendorf, Hamburg, Germany) that were already onboard the ISS [8,9,10]. Additional hardware, including individually packaged sterile pipette tips (Eppendorf), a magnetic separation stand (V&P Scientific, Inc., San Diego, CA, USA) and BEST Swab kits were launched at ambient temperature. All reagents were aliquoted into spaceflight-approved tubes and packaged into one-time-use kits to increase ease-of-use for the crew. These kits include the BEST Swab Kit, BEST Magnetic Bead Kit, BEST Extraction Kit, BEST PCR Kit, BEST Sequencing Kit, and Flow Cells. All kits were assembled at JSC. The BEST Swab kits contained 5 swabs that were autoclaved for 30 min at 120 °C (Texwipe and Idaho Technology), and a 1.5 mL tube of sterile nuclease-free molecular grade water. The BEST Magnetic Bead Kit contained Agencourt AMPure XP beads (Beckman Coulter Genomics) and the BEST Extraction Kit contained QuickExtract™ DNA Extraction Solution (Lucigen). The BEST PCR Kit and Sequencing Kit contained LongAmp^®^ Taq 2X Master Mix (NEB), a SQK-RAB204 16S Barcoding Kit (ONT), 10 mM Tris-HCl 50 mM NaCl, and 5 % polyethylene glycol (PEG) 1.25 M NaCl. Flow cells used were R9.4.1 MIN106 (ONT). The BEST payload was launched on the Orbital ATK OA-9 Cygnus resupply vehicle from Wallops Island, VA on May 21, 2018. With the exception of flow cells and AMPure XP beads, which were stowed and launched at +4 °C, all reagents were stowed and launched at −90 °C. Once berthed to the ISS, all consumables were transferred to the refrigerator (+2 to +8 °C) or freezer (−80 to −90 °C) dewars within the Minus Eighty Degree Laboratory Freezer for the ISS (MELFI), per reagent requirements. All ambient hardware was stored in a half Cargo Transfer Bag (CTB) in the Columbus module of the ISS. During operations, all hardware and reagents were deployed on the Maintenance Work Area (MWA) and secured using Velcro or tape to create a workbench space.

### 2.5. Swab-to-Sequencer Operations Onboard the International Space Station

Three surface swabs and a negative control swab were collected and processed following the culture-independent swab-to-sequencer method as described in Section 2.1 during ISS Expeditions 56 and 57. Sample locations were selected based on quarterly microbial monitoring locations or crew preference. For consistency, crewmembers received onboard pipetting training before starting operations and followed detailed crew procedures, in addition to being guided through ground-based communications. Sequencing was initiated using MinKNOW software version 1.10.23 on a HP ZBook laptop. Sequencing was performed for 48 h and data were downlinked to the ground following completion of the run.

### 2.6. Ground Control Testing of Identical Spaceflight Reagents

Sterile swabs were dipped into 75 μL of whole-cell ZymoBIOMICS Microbial Community Standard (ZYMO RESEARCH, Irvine, CA) or nuclease-free molecular grade water. Swabs were processed on the ground using reagents from the same lot and packaged at the same time as the spaceflight kits. The same spaceflight procedures described above were used for processing.

### 2.7. Nanopore Data Analysis

Fast5 files were basecalled using GPU enabled Guppy Basecalling Software 3.2.8 (ONT). Samples with barcodes were demultiplexed using qcat version 1.1.0 and length filtered using NanoFilt Version 2.5.0 [18] to either 1350-1650 bp for the 16S region or >100 bp for the positive control (−l–1350,--maxlength 1650). Using minimap2 version 2.17-r941 (-ax map-ont) [19], the reads were mapped to a positive control reference of *A. mellifera* or a curated 16S database from either the NCBI Reference Sequence (RefSeq) Database or ZymoBiomics. Primary alignments were extracted using SAMtools version 1.9 and filtered with a MAPping Quality (MAPQ) threshold of 1 [20]. Sequencing statistics were determined using MarginStats (marginStats.py 0.1) [21]. Data were filtered to ≥90% alignment identity. Data visualizations were created in R using the ggplot package, heatmap and correlation network package [22,23,24,25]. Nanopore and Sanger sequencing data have been deposited in the European Nucleotide Archive (ENA) under accession number PRJEB41406.

## 3. Results and Discussion

### 3.1. Swab-to-Sequencer Method for Rapid, In-Situ Microbial Profiling in Extreme Environments

In order to better achieve uniformity between swabs, highly detailed swabbing procedure was developed, including important details about area selection, swabbing technique, and duration (represented by Figure 1A). Early testing indicated that to obtain sufficient DNA, the swab itself needed to be included in the extraction process, as opposed to simply transferring material from the swab to a buffer, followed by performing DNA extraction steps on that buffer. Furthermore, because there is not currently bead beating hardware capable of processing swab tips available for use onboard the ISS, flight-compatible protocols were developed around swab tips that would fit within PCR tubes, utilizing enzymatic and heat-based cell lysis methods. Swabs of various materials were tested such as foam, nylon flocked polyester, polyester bicomponent fiber and a knitted Alpha® polyester swab. The knitted polyester swab worked best and was used in all work described here. With the swab identified, the procedure was developed to ensure that immediately following sample collection, the swab tip was transferred to a PCR tube containing the lysis solution while still at the sampling location. Upon returning from the sampling location, the tube was placed into miniPCR for heating (Figure 1B). This combination of enzymatic and heat lysis was found to be faster, easier to use with less hardware required, and produced a higher yield of DNA, as compared to eluting material from the swab tip into a liquid phase and subsequently processing the liquid with portable bead beating hardware, as was tested onboard the ISS and during our previous method development during the NEEMO 21 mission (Supplementary Table S2) [26].

As the purification of DNA following extraction and PCR is critical for optimal sequencing quality, and as there is not a general use centrifuge onboard the ISS, a paramagnetic bead-based approach was chosen. While ethanol is traditionally used in bead-based purifications, due to its flammability and potential detrimental impacts on the ISS Environmental Control and Life Support Systems (ECLSS), use of ethanol and other alcohols onboard the ISS is tightly controlled and avoided when possible. If used, all alcohol must be maintained under three levels of containment at all times (i.e., multiple glove bags and a glove box or payload hardware specifically designed for containment). As such, a PEG salt solution was determined to be the best alternative for use in all bead clean-ups to purify extracted DNA prior to PCR (Figure 1C) [27,28]. The purified DNA was added to tubes containing master mix and placed in miniPCR for amplification of the full 16S gene (Figure 1D). After a subsequent bead purification (Figure 1E), the PCR product was combined with DNA of a positive sequencing control. To prepare the DNA for sequencing, the nanopore rapid adaptor chemistry was used and the sequencing libraries were added to the flow cell sequencing buffer (Figure 1F). Following previously validated spaceflight procedures, the flow cell was washed twice, the final sample was loaded into the sample loading port on flow cell, and sequencing was initiated from the MinKNOW software installed on the Space Station Computer (SSC) (Figure 1G) [8,10]. Crewmembers were able to use standard air displacement pipettes for all liquid transfer [8].

### 3.2. Ground Validation: Comparison of Current Culture-Dependent to Swab-to-Sequencer, Culture-Independent Methods

**Figure 2:**
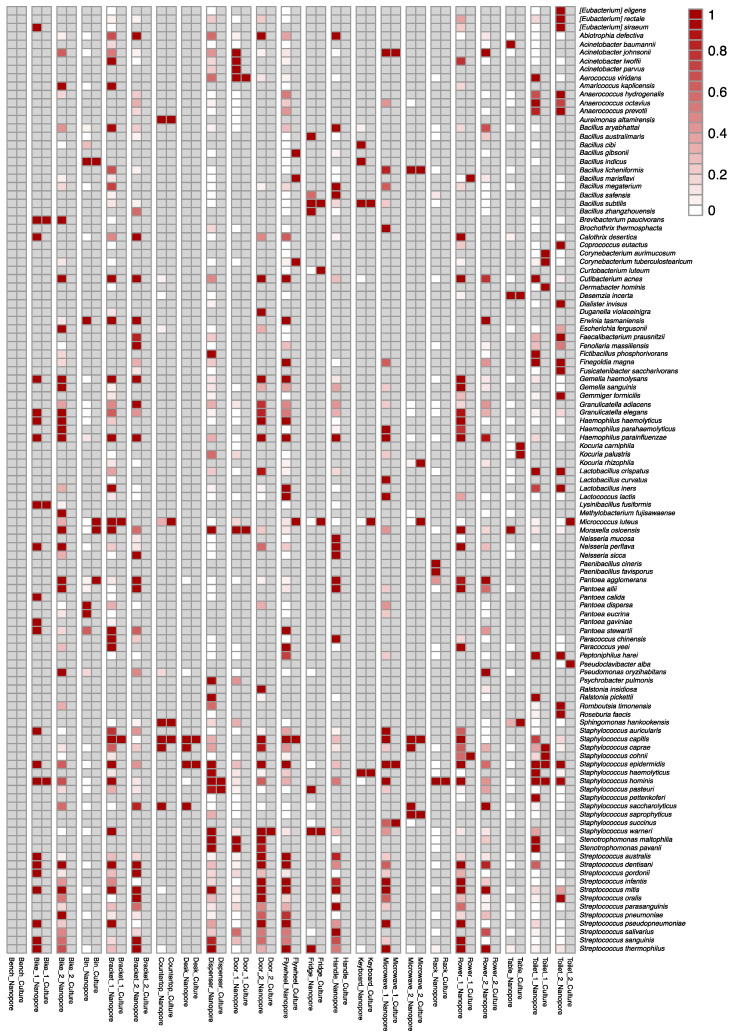
Heatmap of the relative abundance for bacterial species identified using a culture-dependent method as compared to the culture-independent, swab-to-sequencer method. Species less than 0.05% in abundance were excluded. Least abundant to the most abundant is represented from white to dark red with gray as zero abundance. Relative abundance of culture-based isolates was calculated from the colony forming units (CFU). Sample locations referred to in Supplementary Table S2 for more details.

Dual swab collection of the same location allowed for a side-by-side comparison of culture-dependent and culture-independent microbial identification methods. From the 23 swabs used for culturing, 82 culture isolates were observed. Using Sanger sequencing, 60 isolates were identified to the species level, while 21 could not be resolved beyond the genus due to overlapping species identifications. An isolate from bike_1 was unable to grow following subculture from the original community (Supplemental Table S1). In the comparison of culture-based to the culture-independent identifications, 56 out of the 60 species and 21 of the 21 genera were found in corresponding nanopore sequencing data (Figure 2, Supplementary Figure S1). Sequencing data had an average 16S read length of 1,438 bp with a median alignment identity of 90.5% to the NCBI RefSeq database. Among the four unmatched culturable species to sequencing identifications, in three swab locations, toilet_2, microwave_1, and bracket_1, were unable to identify the species *Corynebacterium tuberculostearicum* or *Corynebacterium afermentans*; however, *Corynebacterium* spp. was detected in the nanopore sequencing results (Supplementary Figure S1). Only one culture isolate, *C. tuberculostearicum* from a keyboard swab, was unable to be identified using the culture-independent method.

Consistent with our historical culture data from the ISS using the same media and growth conditions [6], the majority of the culturable isolates were Gram-positive organisms. These included many different species of *Staphylococcus* such as *S. hominis*, *S. epidermidis*, *S. warneri, S. saprophyticus,* and *S. capitis* as well as *Aerococcus viridans*, *C. tuberculostearicum*, and *Micrococcus luteus* (Figure 2). There were four Gram-negative isolates cultured from the swabs that included *Moraxella osloensis*, *Acinetobacter johnsonii*, *Sphingomonas hankookensis,* and *Aureimonas altamirensis*. All of these Gram-positive and Gram-negative species were observed in the corresponding nanopore sequencing results (Figure 2). While culturable bacterial isolates were not detected from swab locations, bike_2 and bracket_2, multiple difficult to culture organisms were present in the nanopore sequencing data (Supplementary Figure S2) including *Streptococcus mitis, Haemophilus parainfluenzae*, and *Abiotrophia defectiva*, which would not have been detectable using our culture methods [29,30,31]. Other abundant species that were not culturable such as *Finegoldia magna*, *Streptococcus thermophilus,* and *Cutibacterium acnes* were also observed throughout the nanopore sequencing data (Supplementary Figure S2). Finally, the bench swab location did not yield any isolates or identifications via nanopore sequencing, demonstrating the ability to characterize a microbially clean surface.

In general, trends from culture-independent sequencing were consistent with published microbiomes of similar sample locations. For example, the organisms in highest abundance from swabbing restroom-related areas (door, toilet seat, and towel dispenser) were characteristic of human-associated bacteria commonly observed in these areas (*A. viridans, M. osloensis*, *M. luteus* or *Lactobacillus crispatus*) (Supplementary Figure S2) [32,33,34]. Furthermore, swabs collected from high touch areas such as handles of kitchen appliances (microwave, fridge), keyboard, and gym equipment (bike, rower, flywheel and brackets) revealed a trend consistent with common skin-associated bacteria such as multiple *Staphylococcus* spp., *Pantoea* spp., *Haemophilus* spp., *Streptococcus* spp. and *Bacillus* spp. (Supplementary Figure S2) [34,35].

By taking advantage of the MinION’s ability to sequence long reads, the entirety of the 16S gene can be sequenced, enabling a substantial improvement in taxonomic classification, often to species level as compared to other culture-independent, next-generation sequencing methods, and Sanger sequencing [36,37,38,39,40]. Oftentimes, species with complex genomes and high similarity cannot be distinguished to the species level with short-read sequencing when using a universal target amplicon [41]. Additionally, bias can occur between identifications dependent on the variable regions used V1-V2 vs. V3-V4 [40]. Full-length 16S sequencing (V1-V9) was reported to generate accurate species-level identifications, first with Pacific Biosciences and in recent years with Nanopore Technology [8,36,42,43,44]. Additionally, our previous method development work onboard the ISS demonstrated accurate species level identifications [8]. Due to the current culture-based molecular short read method used in our ground validation study, *Bacillus* spp., *Pseudomonas* spp., and *Paenibacillus* spp. were only identified up to genus-level for samples handle, rower_2 and rack, respectively (Supplementary Figure S1). Overcoming the limitation of short-read sequencing, nanopore sequencing results of these same samples suggest these organisms could be *Bacillus aryabhattai*, *Pseudomonas oryzihabitans* and *Paenibacillus cineris* (Figure 2).

Although the culture-based data highly parallel the culture-independent sequencing data, expectedly there are differences in outputs between the two methods. Specifically, the abundance of each species differs among the methods. In addition to the presence of unculturable bacteria in the sequencing data, these differences could result from several biases in DNA extraction, PCR efficiency, primer selection, GC content, and data analyses, all of which have been investigated among various sequencing platforms [36,45,46,47,48]. In addition, the impact of bioinformatic methods used to achieve final results is well characterized. New bioinformatic pipelines, such as NanoClust, have been shown to further improve 16S species-level identifications [49]. With recent improvements to nanopore sequencing chemistry, substantial improvements in data quality have been observed. Beyond sequencing chemistry, increasing the amplicon to include the ITS and 23S of the *rrn* operon, as well as the use of unique molecular identifiers has been shown to increase confidence in species classifications and allow for strain level resolution [43,50]. Continued development in both the method and bioinformatic analysis will further increase quality and speed of data output.

The results of our culture-dependent vs. culture-independent sequencing validation study revealed a resemblance among data obtained from two different methods. While there was not complete overlap between the two datasets, the non-detection of *Corynebacterium* spp. from the culture-independent sequencing data would not have altered NASA’s microbial risk assessment. *Corynebacterium* spp. are common in the environment, routinely isolated from the ISS (*C. diphtheriae* has not been isolated from the ISS), and their isolation has not resulted in NASA necessitating remediation. Moreover, molecular data provide a more inclusive description of the environment and are likely to improve NASA’s microbial risk evaluation process. While it is not unexpected to find additional opportunistic pathogens such as *Streptococcus* spp. and *Haemophilus* spp., their presence is not currently part of NASA’s risk assessment, as growth under the conditions provided onboard the ISS has not been noted. With findings from the validation study, the swab-to-sequencer method was further investigated toward operational use in an extreme environment during a NASA analog mission.

### 3.3. Extreme Environment Method Validation during a NASA Analog Mission

**Figure 3:**
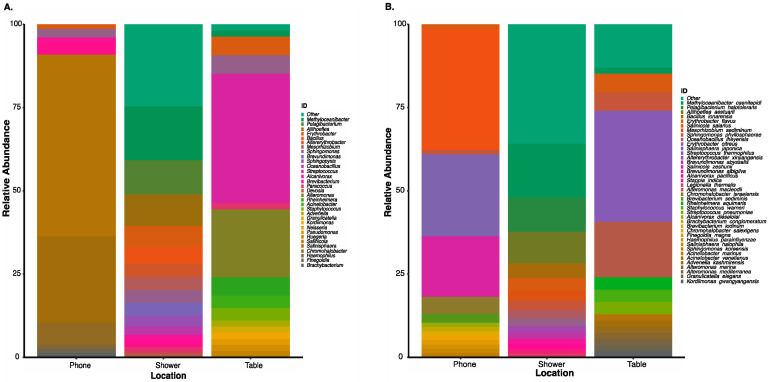
Microbial profile of the top 15 (**A**). genus and (**B**). suggested species level identifications from three distinct locations onboard the Aquarius habitat by NASA Extreme Environment Mission Operations (NEEMO) 22 crew using the culture-independent, swab-to-sequencer method. Alignments performed using minimap2 (-ax map-ont).

Field-testing during NEEMO missions demonstrated that a culture-independent, swab-to-sequencer protocol could be completed successfully in an extreme environment by a crew with varying backgrounds and scientific experience. A highly limited three-hour pre-mission training session and our detailed crew procedures were the extent of guidance provided toward working with the hardware and the swab-to-sequencer method. Once crew and materials were safely within the Aquarius habitat on the ocean floor, the swab-to-sequencer method was evaluated by three different crewmembers on separate mission days. A combination of science team direction and crew preference resulted in swabs being collected from the phone, main dining table, and the shower. The method was implemented as previously described and sequencing successfully completed for each of the three experiments. Total number of flow cell pores available at sequencing initiation were 1375, 1062, and 1098 pores. Average read length and median alignment identity for 16S and positive sequencing control, *A. mellifera*, were 1,484 bp with 91% alignment identity to the NCBI RefSeq database and 730 bp with 94.1% alignment identity to *A.mellifera* reference. The bacteria identified from these three locations contained human-associated bacteria such as *Streptococcus* spp. and *Staphylococcus* spp. (likely *S. pneumoniae and S. warneri)* [51,52], but also contained a large amount of ocean-associated organisms such as *Methyloceanibacter* sp., *Aliihoeflea* sp., *Salinisphaera* spp., *Erythrobacter* spp., and *Oceanobacillus* sp. (Figure 3) [53,54,55,56]. The swab of the shower, located in Aquarius’s wet deck with direct access to the ocean, had the greatest diversity of oceanic microbes. The main table serves as general use for a wide range of activities, and the phone is used frequently to call mission control after diving exercises have been completed. As such, a mixture of human-associated and ocean-associated microbes were observed in these locations. A negative control consisting of reagent kit components processed before and after the mission confirmed that bacteria observed were associated with Aquarius’s microbiome. The success of the sequencing runs and quality of data further establish the robustness of the hardware and reagents in an extreme environment, with limited cold storage capabilities and high atmospheric pressure. Furthermore, the opportunity for field testing of the method during NEEMO provided substantial insight toward improved packaging and labeling of kits, as well as optimization of crew procedures in preparation for application onboard the ISS.

### 3.4. Swab-to-Sequencer Processing Onboard the International Space Station

**Figure 4:**
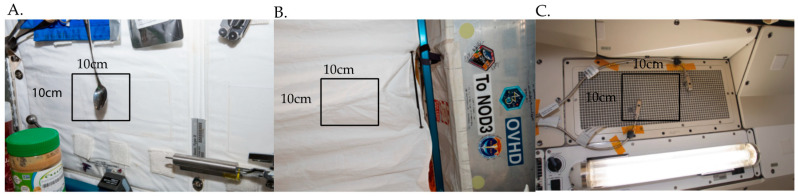
Location of swabbing onboard the International Space Station. (**A**). On July 19, 2018 the Node 1S4 wall adjacent to the crew dining table was swabbed. The area swabbed is directly behind the spoon seen attached to the Node 1S4 interior wall. (**B**). On September 17, 2018 a swab sample was collected from a curtain within the Permanent Multipurpose Module (PMM) that separates the PMM module from the Node 3 module. The area swabbed faces into the PMM. (**C**). On September 24, 2018 a swab sample was collected from an area of the Overhead Aft 7 Air Grid in the Japanese Experiment Module (JEM).

In applying the swab-to-sequencer method onboard the ISS, three samples and a negative control swab were collected and processed, respectively, between two crewmembers during Expeditions 56 and 57. Sampling locations were based on crew preference (Figure 4A) or locations that are included in the quarterly culture-based microbial monitoring of the ISS (Figure 4B,C). The crew performed the swab-to-sequencer method as previously described and observed no issues with implementation in microgravity. Total hands-on crew time from sample collection to sequencing was 3.5 h. Flow cell pore counts were reported by the crew following MinKNOW’s platform quality control (QC) and after sequencing initiation. All flow cells passed QC and had high initial pore counts (Table 1). There was variability in the number of reads obtained from each sample location, ranging from ~43,000 to ~1,700,000 (Table 1). The percentage of reads mapping to 16S references also varied by location and was directly correlated with the total number of reads generated, whereas the percentage of reads that mapped to *A. mellifera* was inversely correlated with the total number of reads (Supplementary Table S3). The disproportion of reads and reads mapping to the 16S gene among surfaces sampled mirrored that of the numbers of colonies arising from differing culturing sites (Table 1, Supplementary Table S3). The majority of return ISS contact slides, used for routine culture-based microbial monitoring, reveal little-to-no growth. However, occasionally a site will give rise to significant numbers of microbes [1], which is consistent with the variation in numbers of reads observed here.

The median alignment identities of 16S and *A. mellifera* alignments across all runs were 91.4% and 91.8%, respectively (Supplementary Table S3). The quality of the *A. mellifera* data from the negative swab after 6 months demonstrates the stability of the sequencing reagents and flow cells in the spaceflight environment (Table 1, Supplementary Table S3). To further confirm the stability of the reagents after 6 months of storage at −80 °C, ground testing was conducted on the identically packaged swab and sequencing kits using ZymoBIOMICS whole-cell microbial community standard. From the ground testing, ~1.7 M reads were generated from the microbial community standard and ~32,000 reads from the negative swab (Table 1). The negative swabs had no alignable reads to the 16S database; however, reads aligned to *A. mellifera* with 91% alignment identity. Median alignment identity for the ZymoBIOMICS microbial community was 91.5% alignment to ZymoBIOMICS reference database, and 92.3% to *A. mellifera* (Supplementary Table S3). All eight of the bacterial species that make up the ZymoBIOMICS Microbial community standard were able to be detected using the swab-to-sequencer method with 6-month-old reagents (Supplementary Figure S3).

### 3.5. Culture-Independent Microbial Profiles of International Space Station Locations 

**Figure 5:**
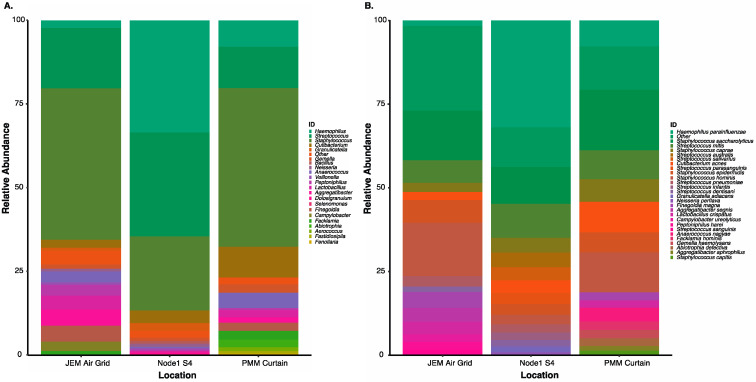
Microbial profile of the top 15 (**A**). genus and (**B**). suggested species level identifications present at three distinct locations onboard the International Space Station detected by a culture-independent swab-to-sequencer method. Alignments performed using minimap2 (-ax map-ont).

Consistent with historical culture-based ISS data, the bacteria identified from the sequencing runs described in Table 1 are associated with the human microbiome [6,57,58] (Figure 5). Sequencing data from onboard the ISS showed increased microbial diversity as well as increased abundances of Gram-negative organisms and Gram-positive anaerobes. This parallels our ground validation study, as well as previous molecular investigations of the ISS microbiome in which samples were processed and sequenced on Earth [12,59,60]. While this work is the first to document the collection of this type of data in situ onboard the ISS, it is not the first to describe the wide-spread abundance of *Staphylococcus*, *Streptococcus*, *Cutibacterium,* and *Haemophilus* across its surfaces [1,6,12,14,61,62]. Routine microbial monitoring cultures returning from the ISS to our lab are dominated by *Staphylococcus*, with *S. hominis and S*. *epidermidis* being the most commonly isolated species. *S. epidermidis* and *S. hominis* were indicated to be present at all sample locations as determined by culture-independent nanopore sequencing (Figure 5). Suggested also at all locations, but in varying relative abundance, were the difficult to culture human-associated *H. parainfluenzae* and *C. acnes* (Figure 5) [57,63]. The Node 1S4 swab of the dining table wall observed a higher abundance of *Streptococcus* species and other oral related species, such as *Neisseria* sp. and *Granulicatelle* sp., as compared to the other two locations [58,64,65], correlating with the astronaut saliva microbiome and previous ISS microbiome study [62,66]. The 15 most abundant suggested species found with this swab-to-sequencer method represented 74.6–88% of total suggested species identified onboard the ISS (Figure 5). Taken together, these data match previous results from our laboratory, and the work of many other investigators, and is clear evidence of crewmember influence on the microbiome of the ISS.

### 3.6. Correlation of Microbial Profiles from the Seafloor to Space Station

**Figure 6:**
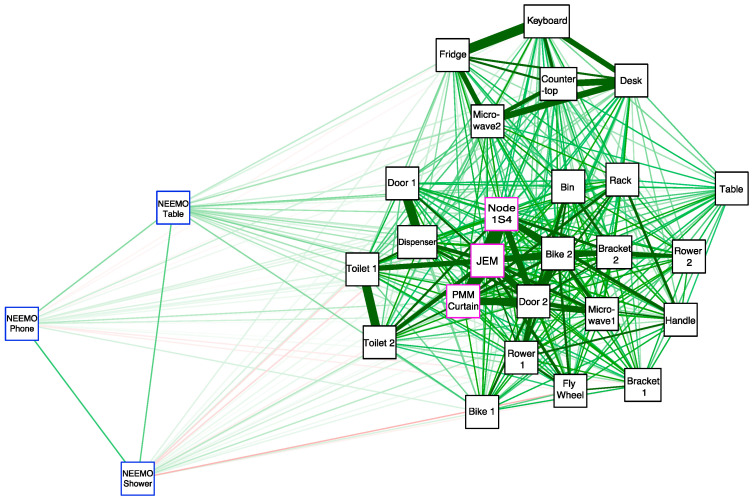
Spearman correlation network showing the associations between swabs collected onboard the ISS (outlined in pink) from the Aquarius Habitat on the seafloor (outlined in blue) and from the Human Health and Performance Laboratory building at the NASA Johnson Space Center (outlined in black). Sample association shown from high to low with green to red color and thick to thin lines.

Detection of unique microbial profiles observed from swabs collected and processed using the swab-to-sequencer method from the Aquarius habitat on the seafloor, a terrestrial office building, and onboard the ISS validated successful application of this method. Spearman correlation confirmed a similarity of swabs from the ISS to ground locations while swabs from NEEMO were distantly related (Figure 6). The distant correlation of samples collected during NEEMO as compared to the ISS and ground swabs was expected due to the abundance of ocean-related organisms observed in the Aquarius habitat. As observed in previous studies comparing the ISS and ground locations, ground swabs of exercise equipment, door handles, and toilet seats were observed to have higher similarity to ISS swabs than other ground swabs [61]. The three swabs from onboard the ISS were observed to be more closely related to each other, likely due to higher abundance of *Haemophilus* and *Staphylococcus* as well as the presence of additional organisms that could also thrive in higher levels of CO_2_ (Figure 5 and Figure 6) [67,68,69]. It is possible that environmental selection pressures associated with the ISS environment, such as elevated levels of atmospheric CO_2_ [70], could be selecting for a greater abundance of capnophilic microorganisms. Future studies with a greater number of ISS swabs are needed to address this hypothesis.

### 3.7. Run Time to 16S Bacterial Identification on International Space Station

**Figure 7:**
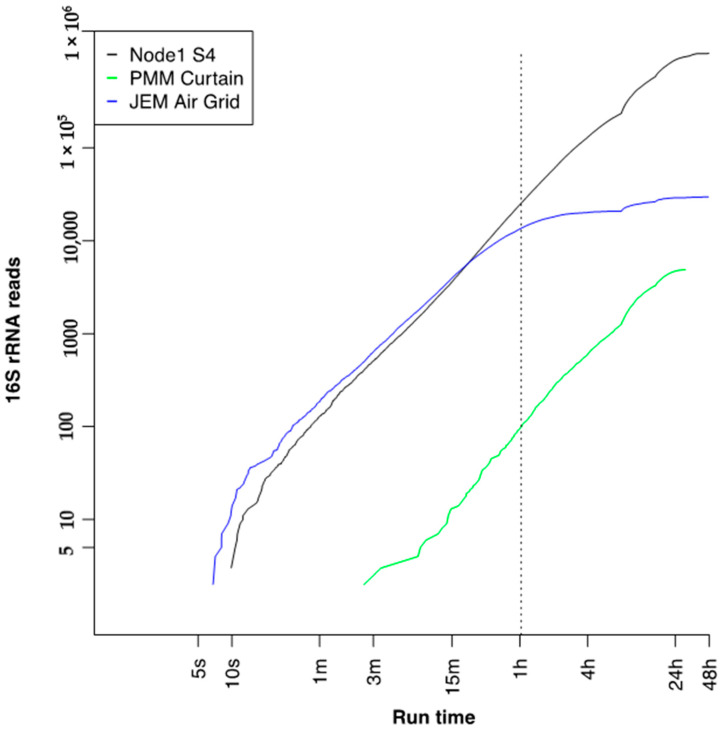
16S reads accumulated over time per three swab-to-sequencer runs onboard the International Space Station. The dotted line represents time when the same top suggested species diversity was recorded as at completion of the run (Supplemental Figure S4).

The acquisition times for reads mapped to the 16S reference database were extracted and observed (Figure 7). For higher throughput runs, Node 1S4 and the Japanese Experiment Module (JEM) Air Grid, the first high-quality reads were generated within 10 s of initiating the run, while a lower throughput run, Permanent Multipurpose Module (PMM) Curtain, required up to 7 min to start generating high quality reads. Due to variations in run throughput, a minimum 1-h run time was needed to generate the same microbial profile as compared to 48 h (Figure 6, Supplementary Figure S4). Assessment of ground swabs also support a 1-h sequencing run time that provided parallel profiles as at the run’s completion (Supplementary Figure S5). Some swabs, likely containing higher biomass, were able to generate high-quality data faster and reduce the total run time needed. However, additional studies are needed to determine an optimal run time to provide the quickest sample-to-answer scenario to support future microbial profiling.

## 4. Conclusions

Technology capable of providing rapid characterization of the environment and human health is critical to extending human space exploration. The method presented here is a validated culture-independent, swab-to-sequencer process for microbial profiling that can be completed entirely onboard the ISS. This method was developed specifically around the many challenges inherent to both the scientific goal (e.g., low biomass swabs and the detection of microbes without traditional culture) and those associated with the ISS environment (e.g., inability to use flammable or toxic reagents, centrifugation, and vortexing, as well as limited crew time). Ground-based laboratory testing confirmed that side-by-side comparison of the current culture-based method to this new culture-independent sequencing method yielded similar microbial detection; however, sequencing data provided a more complete assessment of difficult to culture microbes. Data shown here support this method’s capability to meet part of NASA’s microbial monitoring requirement by identifying bacterial organisms on ISS; however, work is in progress to further validate this method with additional comparative samples and extend it toward identification of fungal organisms as well as toward quantification. To accomplish true in situ independence, advanced offline analytics will also be required. Future work will demonstrate rapid data analysis onboard the ISS in support of a fully autonomous sample-to-answer system. This swab-to-sequencer method, along with on-orbit data analysis, is adaptable to extend beyond environmental microbial profiling and to potentially aid in infectious disease diagnostics to enable human exploration of the Moon and on to Mars.

## Figures and Tables

**Table 1:** Nanopore sequencing date, sample location, total FAST5 reads generated and flow cell pore counts onboard the International Space Station and ground controls.

Date	Location	Total Reads (Fast5)	QC Pore Count	Sequencing Pore Count
19 July 2018	Node 1S4 Dining Table Wall	1,660,150	1646	1635
17 September 2018	Permanent Multipurpose Module (PMM) Curtain	172,891	1610	1574
25 September 2018	Japanese Experiment Module (JEM) air grid	92,106	1530	1002
7 November 2018	Flight Negative Swab	42,880	1505	1486
23 October 2018	Ground Negative Swab	31,970	1613	1589
16 November 2018	Ground Positive Control	1,689,464	1613	1589

## Data Availability

Publicly available data sets were analyzed in this study. The data can be found in the European Nucleotide Archive under accession number PRJEB41406.

## Supplementary Materials

The following are available online at https://www.mdpi.com/2073-4425/12/1/106/s1, Table S1: Description of sampling locations and DNA concentrations for swabs collected from the NASA Johnson Space Center’s Human Health and Performance Laboratory in Houston, Texas, Table S2: Ground swab DNA extraction yields comparison between Claremont Bio OmniLyse^®^ Cell Lysis Device and Lucigen QuickExtract^TM^, Table S3: Sequencing run metrics for total reads mapped to 16S and *A. mellifera* positive control from sequencing runs onboard the International Space Station and ground controls, Figure S1: Heatmap of the relative abundance for bacterial genus identified using culture-dependent method as compared to the culture-independent, swab-to-sequencer method, Figure S2: The top 15 most abundant suggested species level identifications from ground swabs collected from the NASA Johnson Space Center’s Human Health and Performance Laboratory using the culture-independent, swab-to-sequencer nanopore sequencing method, Figure S3: Actual vs. theoretical percentage of reads assigned to ZymoBIOMICS Microbial Community Standard reference genomes based on alignment using minimap2 (-ax map-ont), Figure S4: Top 15 suggested species level identifications from three distinct locations onboard the International Space Station detected within 1 h and 48 h of nanopore sequencing using the culture-independent swab-to-sequencer method, Figure S5: Top 5 suggested species level identifications from ground swabs collected from the NASA Johnson Space Center’s Human Health and Performance Laboratory at 1 h vs run completion using the culture-independent, swab-to-sequencer method.
