# Supplementary material for: Real-Time Culture-Independent Microbial Profiling Onboard the International Space Station Using Nanopore Sequencing
Source: Genes (Basel). 2021 Jan 16;12(1):106. doi: 10.3390/genes12010106 (PMC7830261; doi:10.3390/genes12010106)
Supplement: Supplementary file 1 [file genes-12-00106-s001.pdf]

Supplementary Materials

Table S1. Description of sampling locations and DNA concentrations for swabs collected from NASA Johnson Space Center's Human Health and Performance Laboratory in Houston, Texas.

Name	Descriptions of sample	Location	DNA (ng/ul)
Bench	Back of leg press exercise bench	High Bay Exercise Room, North Wing	<0.01
Bike-1	Bike handle bars, swab 1	Rm 1229, North Wing	0.137
Bike-2	Bike handle bars, swab 2	Rm 1229, North Wing	0.276
Bin	Plastic storage bin	Rm 1229, North Wing	0.132
Bracket-1	Harness bracket	Rm 1229, North Wing	0.816
Bracket-2	Harness bracket	Rm 1229, North Wing	0.154
Countertop	Breakroom countertop	Rm 1100, South Wing	0.14
Desk	Desk countertop	Rm 1108D, South Wing	0.212
Dispenser	Paper towel dispenser sensor area	Rm 11RW, South Wing	0.114
Door-1	Women restroom door handle, swab 1	Rm 11RW, South Wing	<0.01
Door-2	Women restroom door handle, swab 2	Rm 11RW, South Wing	<0.01
Flywheel	Handle on Flywheel exercise device	Rm 1229, North Wing	0.086
Fridge	Refrigerator door handle	Rm 1100, South Wing	0.17
Handle	Handle on leg press exercise device	High Bay Exercise Room, North Wing	0.272
Keyboard	Computer keyboard	Rm 1112D, South Wing	0.164
Microwave-1	Microwave door handle, swab 1	Rm 1100, South Wing	0.102
Microwave-2	Microwave door handle, swab 2	Rm 1100, South Wing	0.206
Rack	Weight Rack	High Bay Exercise Room, North Wing	0.124
Rower-1	Handle on Rower exercise device, swab 1	Rm 1229, North Wing	0.098
Rower-2	Handle on Rower exercise device, swab 2	Rm 1229, North Wing	0.318
Table	Breakroom countertop	Rm 1100, South Wing	<0.01
Toilet-1	Toilet seat, swab 1	Rm 11RW, South Wing	0.197
Toilet-2	Toilet seat, swab 2	Rm 11RW, South Wing	0.164

Note: the number following the sample name is the replicate of the sample.

Table S2. Ground swab DNA extraction yield comparisons between Claremont Bio OmniLyse® Cell Lysis Device and Lucigen QuickExtract™. Extracted DNA was measured using Qubit dsDNA high sensitivity kit (Thermo Fisher Scientific). Swabs were collected from parallel sample locations and identical concentrations of ZymoBIOMICS Microbial Community Standard (ZYMO Research) was used prior to DNA extraction.

**Swab Location**	**QuickExtract™ and Heat (ng/μl)**	**OmniLyse® Cell Lysis Device (ng/μl)**
Toilet Seat	0.0456	<0.0005
Bathroom Door	<0.0005	<0.0005
Conference Room Table	0.0640	<0.0005
Keyboard	0.1630	<0.0005
Coffee Machine	0.0908	<0.0005
Microwave Door	0.4480	<0.0005
Negative Swab	<0.0005	<0.0005
ZymoBiomics Microbial Community Standard	12.8	4.2

Table S3. Sequencing run metrics for total reads mapped to 16S and *A. mellifera* positive control from sequencing runs onboard the International Space Station and ground controls.

Location	Sample Type	Mapped Reads	Mean Read Length (bp)	Median Alignment Identity (%)
Node 1S4 Dining Table Wall	16S	234,523	1572	91.4
	A.mellifera	46,772	725	92.2
Permanent Multipurpose Module (PMM) Curtain	16S	872	1578	91.2
	A.mellifera	5,179	732	91.8
Japanese Experiment Module (JEM) air grid	16S	10,852	1583	91.6
	A. mellifera	3,886	730	92.1
Flight Negative Swab	16S	0	NA	NA
	A.mellifera	15,045	722	91
Ground Negative Swab	16S	0	NA	NA
	A.mellifera	1,276	716	92.2
Ground Positive Control	16S	59,475	1568	91.5
	A.mellifera	226	727	92.3

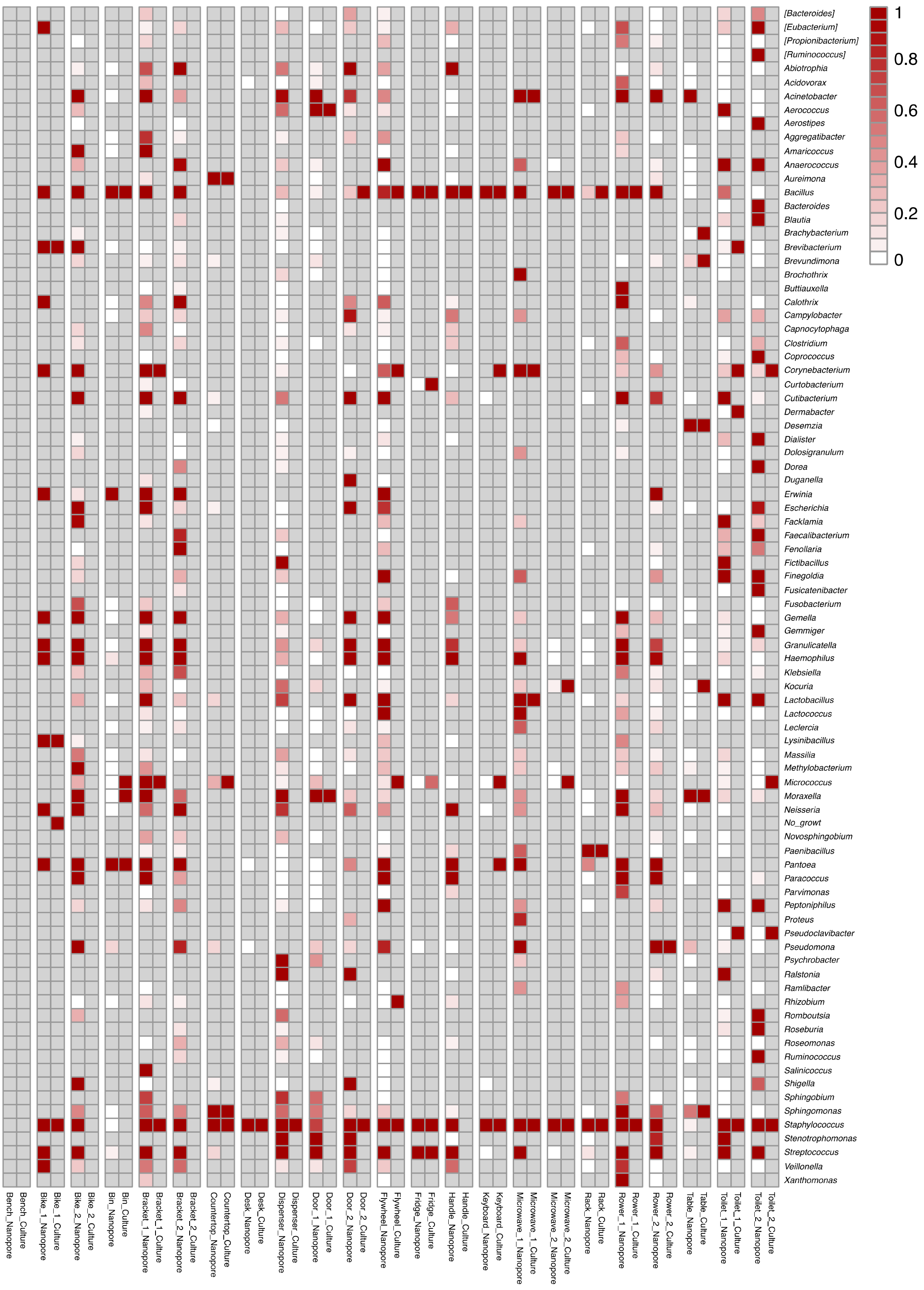

Figure S1. Heatmap of the relative abundance for bacterial genus identifications using culture-dependent method as compared to the culture-independent, swab-to-sequencer method. Genus less than 0.02% in abundance are excluded. The least abundant to the most abundant is represented from white to dark red with gray as zero abundance. Relative abundance of culture-based isolates was calculated from the colony forming units (CFU). Sample locations referred to Table S2 for more details.

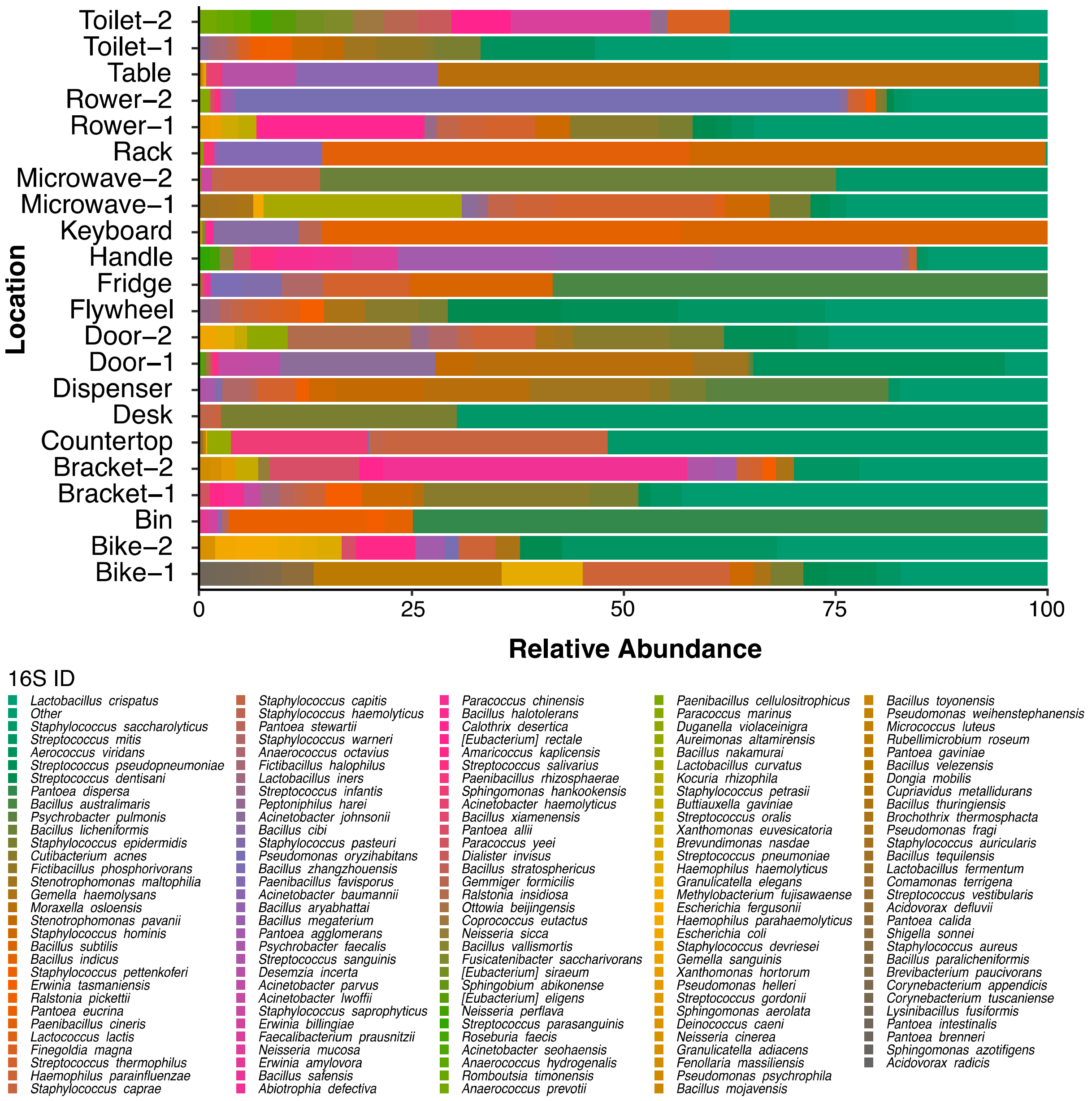

Figure S2. The top 15 most abundant suggested species level identifications from ground swabs collected from the NASA Johnson Space Center's Human Health and Performance Laboratory using the culture-independent, swab-to-sequencer nanopore sequencing method. Alignments performed using minimap2 (-ax map-ont).

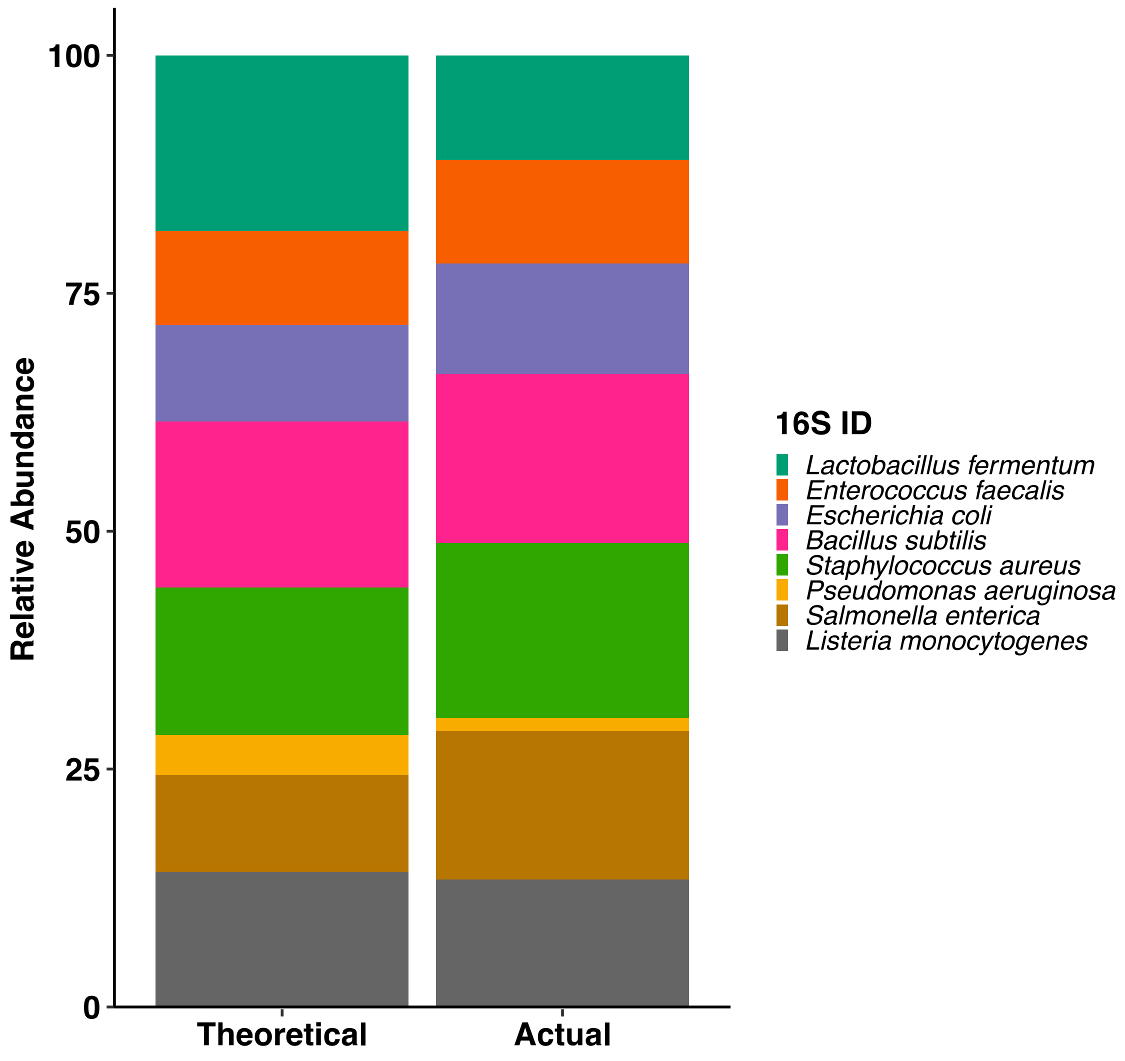

Figure S3. Actual vs. theoretical percentage of reads assigned to ZymoBIOMICS Microbial Community Standard reference genomes based on alignment using minimap2 (-ax map-ont). Nanopore sequencing library was prepared using whole-cell ZymoBIOMICS Microbial Community Standard with reagents that were prepared and packaged in parallel to International Space Station reagents and stored at -90°C for > 6 months.

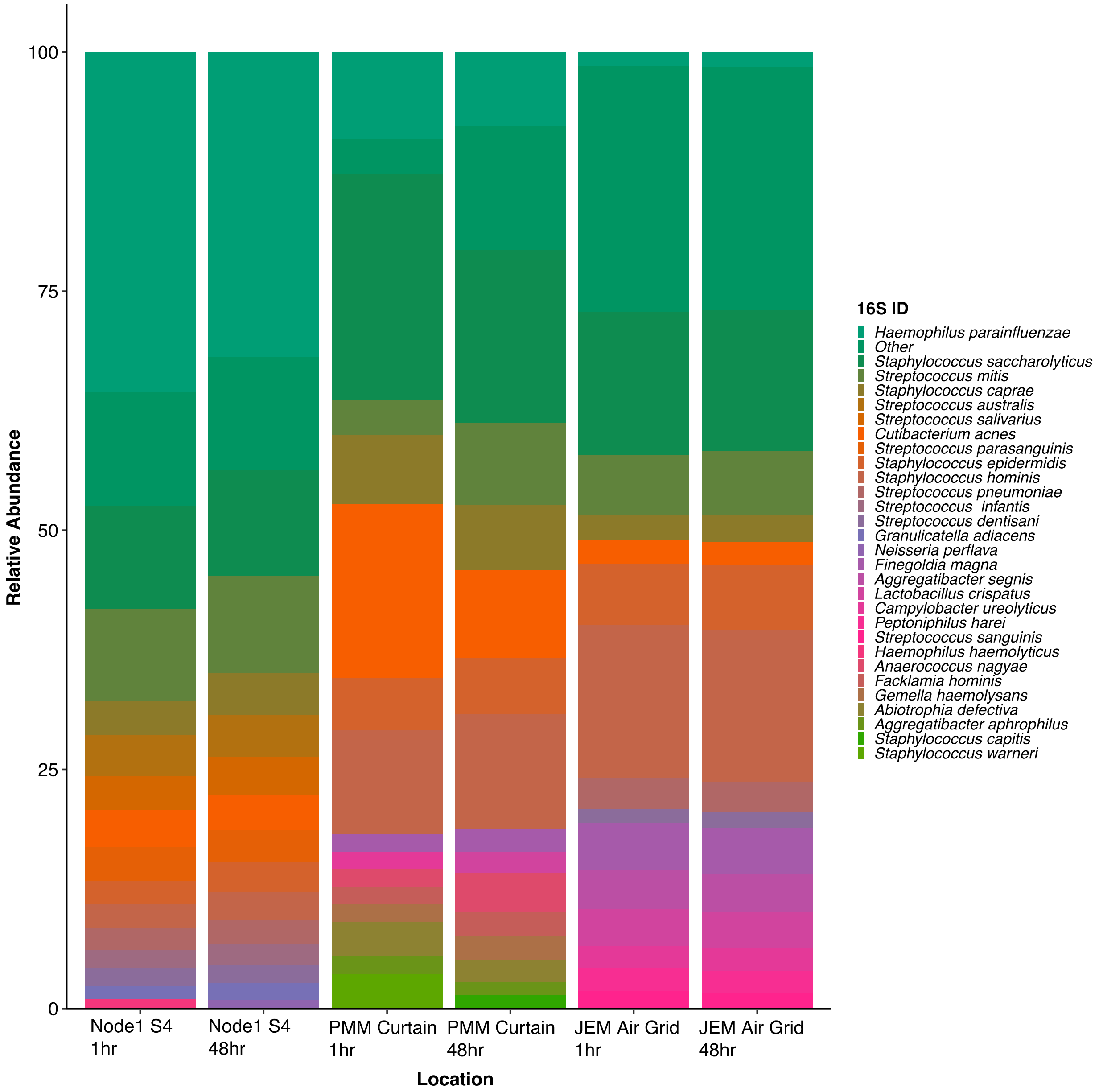

Figure S4. Top 15 suggested species level identifications from three distinct locations aboard the International Space Station detected within 1 hour and 48 hours of nanopore sequencing using culture-independent swab-to-sequencer method. Colors represent same identification throughout.

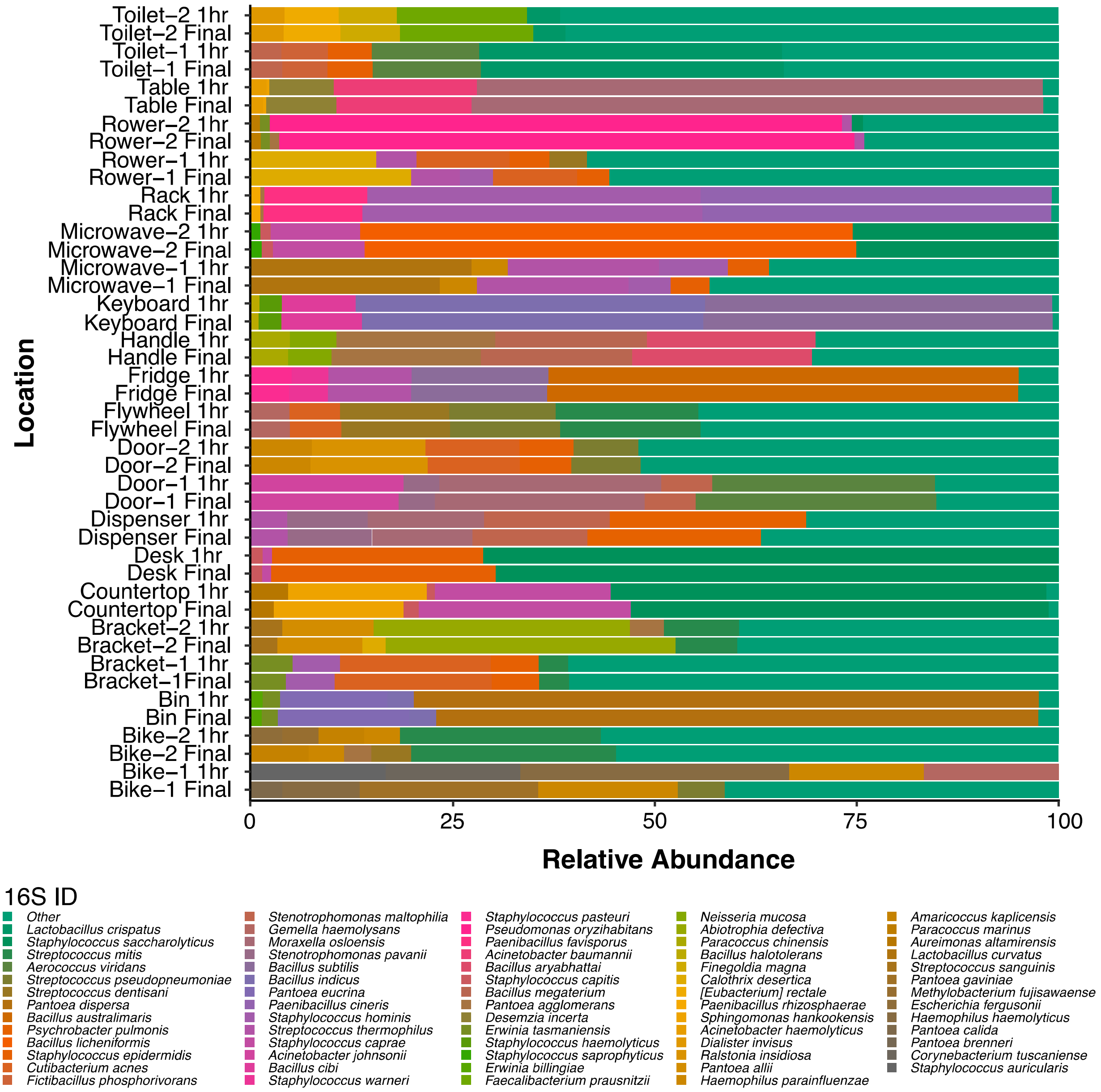

Figure S5. Top 5 suggested species level identifications from ground swabs collected from NASA Johnson Space Center's Human Health and Performance Laboratory at 1 hours vs run completion using the culture-independent, swab-to-sequencer method. Alignments performed using minimap2 (-ax map-ont).
